# Serum microRNA Biomarkers for Detection of Non-Small Cell Lung Cancer

**DOI:** 10.1371/journal.pone.0032307

**Published:** 2012-02-28

**Authors:** Patrick T. Hennessey, Tiffany Sanford, Ashish Choudhary, Wojciech W. Mydlarz, David Brown, Alex Tamas Adai, Michael F. Ochs, Steven A. Ahrendt, Elizabeth Mambo, Joseph A. Califano

**Affiliations:** 1 Department of Otolaryngology-Head and Neck Surgery, Johns Hopkins Medical Institutions, Baltimore, Maryland, United States of America; 2 Asuragen, Inc., Austin, Texas, United States of America; 3 MiRNA Therapeutics, Inc., Austin, Texas, United States of America; 4 Department of Oncology, Johns Hopkins Medical Institutions, Baltimore, Maryland, United States of America; 5 Division of Biostatistics and Bioinformatics, Johns Hopkins Medical Institutions, Baltimore, Maryland, United States of America; 6 Division of Surgical Oncology, University of Pittsburgh Medical Center, Pittsburgh, Pennsylvania, United States of America; 7 Milton J. Dance Head and Neck Center, Greater Baltimore Medical Center, Baltimore, Maryland, United States of America; Johns Hopkins School of Medicine, United States of America

## Abstract

Non small cell lung cancer (NSCLC) is the leading cause of cancer-related mortality world-wide and the majority of cases are diagnosed at late stages of disease. There is currently no cost-effective screening test for NSCLC, and the development of such a test is a public health imperative. Recent studies have suggested that chest computed tomography screening of patients at high risk of lung cancer can increase survival from disease, however, the cost effectiveness of such screening has not been established. In this Phase I/II biomarker study we examined the feasibility of using serum miRNA as biomarkers of NSCLC using RT-qPCR to examine the expression of 180 miRNAs in sera from 30 treatment naive NSCLC patients and 20 healthy controls. Receiver operating characteristic curves (ROC) and area under the curve were used to identify differentially expressed miRNA pairs that could distinguish NSCLC from healthy controls. Selected miRNA candidates were further validated in sera from an additional 55 NSCLC patients and 75 healthy controls. Examination of miRNA expression levels in serum from a multi-institutional cohort of 50 subjects (30 NSCLC patients and 20 healthy controls) identified differentially expressed miRNAs. A combination of two differentially expressed miRNAs miR-15b and miR-27b, was able to discriminate NSCLC from healthy controls with sensitivity, specificity, positive predictive value (PPV) and negative predictive value (NPV) of 100% in the training set. Upon further testing on additional 130 subjects (55 NSCLC and 75 healthy controls), this miRNA pair predicted NSCLC with a specificity of 84% (95% CI 0.73–0.91), sensitivity of 100% (95% CI; 0.93–1.0), NPV of 100%, and PPV of 82%. These data provide evidence that serum miRNAs have the potential to be sensitive, cost-effective biomarkers for the early detection of NSCLC. Further testing in a Phase III biomarker study in is necessary for validation of these results.

## Introduction

Lung cancer is the leading cause of cancer-related mortality world-wide, and was responsible for 1.38 million deaths in 2008 [1]. Smoking is the primary risk factor for lung cancer, and it is estimated that 20.8% of the American adults are active smokers [2]. Currently there is no validated, cost-effective screening test that reliably provides a diagnosis of lung cancer. The development of such a test is a public health imperative since early diagnosis and treatment of lung cancer is associated with up to a 92% 5-year survival [3]. Because lung cancer does not usually become clinically apparent until it reaches an advanced stage, greater than 75% of lung cancers are diagnosed after the disease is already locally advanced or metastatic [4]. Due to the substantial survival advantage to early detection, there have been extensive efforts to detect lung cancer at an early stage. The Early Lung Cancer Action Project (ELCAP) [5] and the National Lung Cancer Screening Trial (NLST) [4] are prospective studies that screened symptom-free high-risk smokers using low dose computed tomography (CT) and preliminary results show increased ability to detect early stage, potentially curable lesions [3]. The NLST was stopped early in November of 2010 after the preliminary data revealed a 20.3% decrease in lung cancer deaths in the CT screening arm of the trial [4], [6]. However, the high false positive rate of 96.4% observed in the low dose CT group is likely to hinder the adoption of CT scans in population screening [6]. In addition, questions about the cost-effectiveness of CT-based screening for lung cancer remain unanswered [7], [8], [9], [10], [11]. Also, there is some concern that repeated exposure to low dose CT scans may expose patients to potentially harmful levels of radiation that could result in more cancers [12]. Although CT scanning can identify lesions suspicious for lung cancer, tissue diagnosis is the only way to determine if a lung lesion is cancerous. A meta-analysis of 7 lung cancer screening studies evaluated low dose helical CT scanning as a screening test for lung cancer and found that 14–55% of high-risk patients, with age ≥40 and ≥20 pack year smoking history, who had a suspicious lung lesion on a screening CT were ultimately found to have benign lung lesions after undergoing an invasive procedure for tissue diagnosis [13]. This high rate of invasive procedures for benign disease underscores the necessity for additional screening modalities that can potentially reduce the number of patients who undergo invasive procedures unnecessarily.

In addition to the large trials investigating the efficacy of CT screening for lung cancer, numerous groups are actively investigating the possibility of blood-based biomarkers for non-small cell lung cancer (NSCLC) [14]. Circulating biomarkers are attractive for cancer screening since they are blood-based tests that are minimally-invasive, relatively low-cost and easily repeatable. Serum microRNAs (miRNAs) are attractive candidates to be used as cancer biomarkers. Serum miRNA can be reliably isolated from serum and have been shown to be highly stable, even under harsh conditions such as multiple freeze-thaw cycles and changes in pH [15]. Recent promising studies suggest that plasma miRNAs could be a useful step in the screening process for lung cancer, and for deciding which patients to further screen by CT scan [16], [17], [18]. In an effort to develop non-invasive biomarker assays that can be used for early detection of lung cancer, we evaluated the expression of miRNA extracted from serum obtained from pre-treatment NSCLC patients and cancer-free healthy subjects, to identify miRNA-based biomarkers that are capable of distinguishing between these groups.

## Methods

### Sample Collection

Samples used in this study were collected at the University of Rochester Medical Center by Dr. Stephen Ahrendt as part of a lung cancer screening study and at The Johns Hopkins Hospital as part of a head and neck cancer screening protocol. Clinical information was also collected for each patient at the time of blood collection. Written informed consent was obtained from all patients before enrollment in the studies, and the studies were approved by the University of Rochester Medical Center Research Subjects Review Board and the Johns Hopkins Hospital Institutional Review Board, respectively. Blood from NSCLC patients, and healthy donors was collected into BD Vacutainer® Plus plastic serum tubes and processed into serum. For all cancer patients, blood was collected at the time of diagnosis but prior to tumor resection or treatment. The serum was immediately stored at −80°C until time of use. After the collection process was completed, all of the records were de-identified to protect patient confidentiality. Cohorts were compiled retrospectively from these large collections of serum samples in an effort to compile age and gender matched cohorts with similar smoking history and with early stage tumors for cancer patients (Table 1). Patients were defined as having a history of smoking if they had a history of consistently smoking for at least one year. A two tailed Fisher's exact test was used to determine the associations between All serum samples are maintained in the tissue bank of the Johns Hopkins Hospital Division of Head and Neck Cancer Research, using a web database application provided by The Johns Hopkins Hospital Department of Oncology's Research Information Technology Systems (RITS) (https://www.rits.onc.jhmi.edu/). Specimens were shipped to Asuragen, Inc., Austin TX for RNA isolation and evaluation of miRNA and data analysis.

**Table 1:** Demographic and histopathologic data for serum samples.

	Normal Training Set	NSCLC Training Set	Normal Test Set	NSCLC Test Set
**N = **	20	30	75	55
**Mean Age (years)**	54.8	66.5	65.7	68.2
**Median Age (years)**	54	66	66	68
**Age Range (years)**	20*–75	56–88	38–85	48–85
**Ethnicity**				
**% Caucasian**	85	93.3	80.1	94.5
**% Africian American**	10	3.3	17.3	5.5
**% Hispanic**	5	0	1.3	0
**% Unknown**	0	3.4	1.3	0
**% Female**	45	45	33.3	43.6
**%Smoking History**	45	80	87	100
**% Adenocarcinoma**	n/a	66.7	n/a	54.5
**% Squamous cell carcinoma**	n/a	33.3	n/a	45.5
**% Stage I Tumor**	n/a	33.3	n/a	60
**% Stage II Tumor**	n/a	30	n/a	24
**% Stage III Tumor**	n/a	33.6	n/a	12
**% Stage IV Tumor**	n/a	0	n/a	4

NSCLC, non-small cell lung cancer; Normal, cancer free, healthy controls.

*Only one healthy control donor was under 35 years.

### Extraction of Serum RNA

Serum RNA (0.5 ml) was extracted by the Asuragen Pharmacogenomics Services Group using the mirVana PARIS Kit (Ambion, Austin, TX), according to the manufacturer's instructions. After the organic extraction, the aqueous phase was loaded onto the columns provided in the kit. RNA was washed and extracted as per manufacturer's instructions. RNA was quantified using the NanoDrop 1000 (NanoDrop, Wilmington, DE) and stored at −80C. RNA yields obtained were typically 300–500 ng/mL of serum.

### miRNA Quantification by RT-qPCR

We used TaqMan RT-qPCR assays (Applied Biosystems, Carlsbad, CA) to examine the expression of 181 miRNAs in serum RNA of 50 subjects, (30 patients with NSCLC, and 20 cancer-free, healthy subjects). All reagents, primers and probe were purchased from Applied Biosystems. Reverse transcription (RT) was performed in 10 uL reactions, each containing 1× RT buffer (Invitrogen, Carlsbad, CA), 250 uM each dNTPs (GE Healthcare, Piscataway, NJ), 2 uL TaqMan RT primer (Applied Biosystems, Carlsbad, CA), 4 units of RNAse inhibitor (Promega, Madison, WI), 10units MMLV-RT (Invitrogen, Carlsbad, CA), and ∼1 ng of RNA per reaction. The reaction mixture was incubated at 16°C for 30 min, 42°C for 1 hr and 85°C for 5 min. qPCR reactions were performed using the 384-well ABI Prism 7900 HT Sequence detection system (Applied Biosystems, Carlsbad, CA). For miRNA screening, one RT and qPCR reaction per sample was performed, whereas for miRNA verification assays post screening, two RT reactions followed each by one qPCR were performed for each of the 130 samples used for validation. Each qPCR was performed in 15 uL reactions containing 1× Platinum Taq buffer (Invitrogen, Carlsbad, CA), 5 mM MgCl_2_ (Invitrogen), 250 uM dNTPs, 2 uL TaqMan microRNA assay primer/probe mix (Applied Biosystems, Carlsbad, CA), 1× ROX, (0.5 units Platinum Taq (Invitrogen, Carlsbad, CA), and 2 uL cDNA from the RT reaction.

### Data Analysis

To identify candidate biomarkers for distinguishing NSCLC from healthy controls, we first calculated the ΔCt value matrix for each sample by subtracting the threshold cycle number (Ct) value for one miRNA from the Ct value of another miRNA in the same sample. The ΔCt matrix approach of considering the set of all differentially expressed miRNA pairs though computationally burdensome, but has the advantage of obviating the need for invoking explicit normalizers. For the 181 miRNAs analyzed per sample, each sample vector yielded 16290 elements (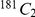), herein referred to as miRNA “diffpairs”.[19], [20] We then computed the unequal variance t-test p-values and the AUC for the ROC curve for each of the diffpairs. The cutoff point for each ΔCt was selected to maximize the sum of sensitivity and specificity. Candidate miRNA pairs for verification in another sample set were further selected based on a sensitivity and specificity of at least 80% each. These criteria produced a total of 140 candidate miRNA diffpairs (Figure S1). The cutoff point used for each miRNA diffpair in the training set was applied in the validation dataset.

## Results

**Figure 1:**
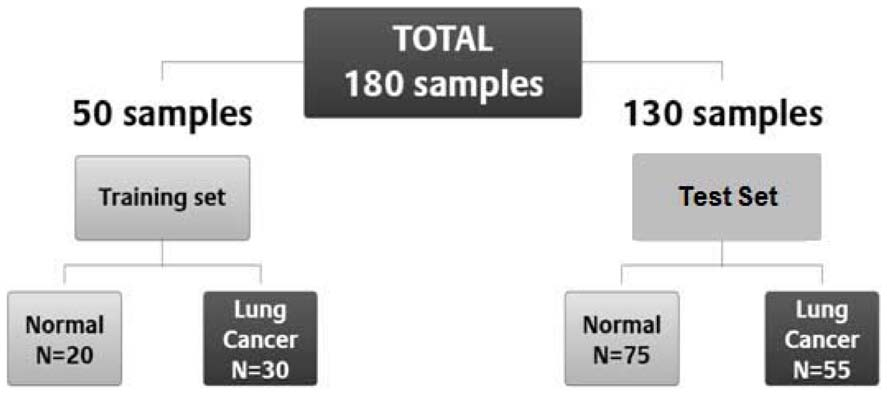
Retrospective study design used to identify miRNA that could distinguish healthy controls (normal) from lung cancer patients.

In order to identify differentially expressed miRNA in NSCLC, we initially screened sera from 16 NSCLC patients and 20 healthy donors for the expression of 328 miRNA using RT-qPCR. To avoid false detection, we first eliminated all miRNAs that were undetected after 40 cycles of qPCR in all samples, leaving only 181 miRNAs. Consequently, we limited further screening of sera from 30 NSCLC patients and 20 healthy subjects to only the 181 miRNAs that were expressed at or below 40 cycles. Patients were matched for age, gender, and smoking history. A two-tailed Fisher's exact tests used to analyze the groups. The only statistically significant association was between cancer and smoking (p = 0.015). Attempts were made to balance the representation of squamous cell carcinomas and adenocarcinomas. The majority of the training set samples (66.7%) were adenocarcinoma, while 33.3% were squamous cell carcinoma. The demographic characteristics of the 30 NSCLC patients and 20 healthy patients with no history of cancer are shown in Figure 1 and Table 1. Although the age range was 20–75 years in the healthy controls, only one donor was below 35 years old.

**Figure 2:**
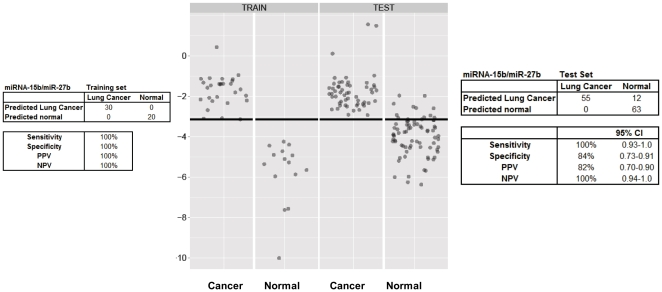
Differential expression of miRNA diffpair miR-15b/miR27-b in sera from healthy donors (normal) and from lung cancer patients (cancer). Differential expression values were calculated as a difference of the Ct values for the two miRNAs. The threshold indicated by the horizontal line was selected to maximize the sum of sensitivity and specificity as described in data analysis. The sensitivity, specificity, positive predictive value (PPV), and negative predictive value (NPV) obtained using the differential expression of the 2 miRNA displayed as tables for the training set (on the right) and the test set (on the left).

**Figure 3:**
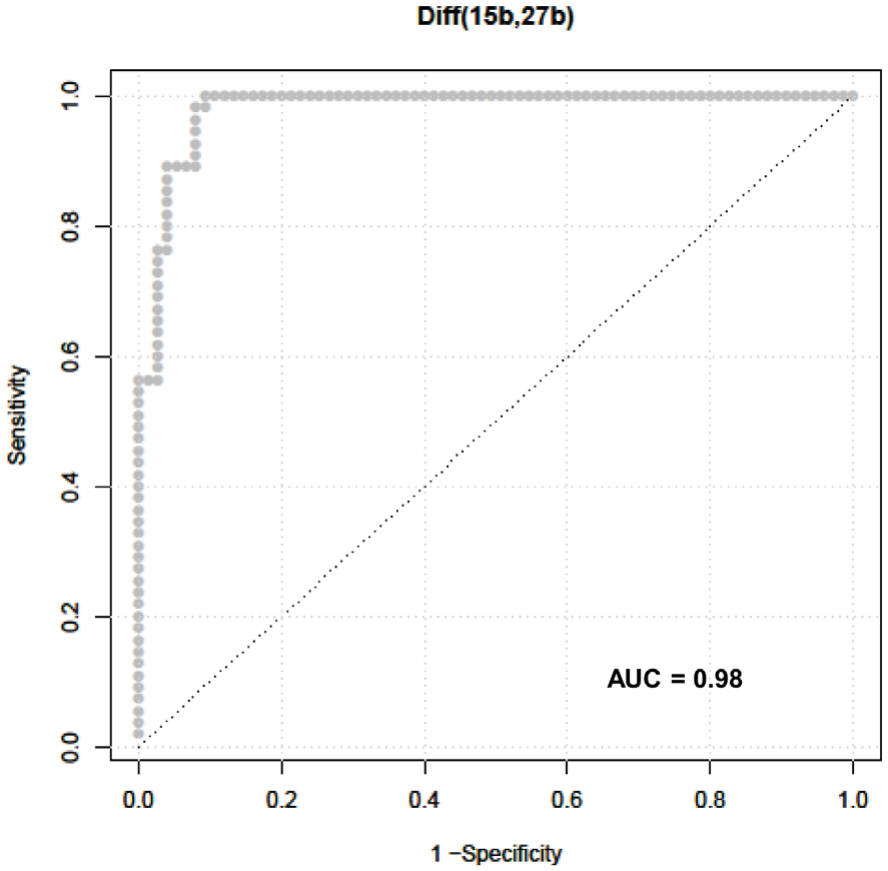
Receiver Operating Characteristic (ROC) plot for the diffpair miR-15b/miR27b from the test set.

Using the differentially expressed miRNA pair-wise data analysis described above, the training set data on 181 miRNAs yielded 16290 diff pairs, of which 140 candidate miRNA pairs distinguished NSCLC from healthy controls with a sensitivity and specificity of at least 80% each (See Figure S1). Several miRNA pairs involving miRNAs-106a, miR-15b, miR-27b, miR-142-3p, miR-26b, miR-182, 126#, let7g, let-7i and miR-30e-5p exhibited a negative predictive value (NPV) and a positive predictive value (PPV) of 100% (Table 1), indicating these miRNAs as putative biomarker candidates for lung cancer diagnosis. The 140 candidate miRNA pairs represented a total of 26 unique miRNAs. Consequently, RT-qPCR of the 26 miRNAs was performed on serum RNA from an additional 55 NSCLC patients (60% Stage I, 24% Stage II, 12% Stage III and 4% Stage IV), and from 75 cancer-free, healthy controls. The demographic characteristics of the 55 NSCLC patients and 75 healthy patients with no history of cancer are shown in Figure 1 and Table 1. A two-tailed Fisher's exact tests used to analyze the groups. The only statistically significant association was between cancer and smoking (p = 0.005). Differential expression of the candidate biomarker miRNA pairs from the training set (Figure S1) was examined in the test set using the same cut-off point as was applied in the training set. The results yielded 5 candidate biomarkers with a sensitivity and specificity of at least 75% (Table 2). All the 5 candidate miRNA pairs shown in Table 2 were significantly differentially expressed between NSCLC and healthy controls, as indicated by the p-values <0.001. Differential expression of the miRNA pair miR-15b/miR-27b is shown in Figure 2 for both the training set and the test set. The distribution of this miRNA pair was spread over a broader range (>4 Cts) in the healthy controls, while the distribution in the NSCLC samples was narrower with a range of ∼2 Cts. The area under curve (AUC) of the receiver operating characteristic (ROC) plot (Fig. 3) for this miRNA pair was 0.98 for the test data, with a sensitivity and specificity of 100% in the training set (Figure S1) and a sensitivity of 100% (95% CI; 0.93–1.0) and specificity of 84% (95% CI 0.73–0.91) in the test set (Table 2 and Figure 2). The second ranking miRNA pair involved miR-15a and miR-27b, with a sensitivity of 87% and specificity of 93% in training set, while its sensitivity and specificity in test set was 94% and 75% respectively (Table 2). Several of the miRNA pairs in the training set had suboptimal performance in the test set with either sensitivity and/or specificity less than 75%. The top candidate miRNA pair (miR-15b and 27b) distinguished NSCLC from healthy controls with a NPV of 100% and a PPV of 82% in the test set (Table 2). These findings show the potential of serum-based miRNA as screening biomarkers for lung cancer.

**Table 2:** Differential miRNA expression in sera from NSCLC patients (cancer) and healthy controls (normal) in test set.

miRpair	PPV	NPV	SENS	SPEC	p-value
Diff(15b,27b)	82%	100%	100%	84%	3.70E-25
Diff(15a,27b)	73%	95%	94%	75%	4.01E-16
Diff(142-3p,27b)	73%	89%	87%	76%	2.36E-13
Diff(15b,301)	89%	83%	75%	93%	2.17E-16
Diff(27b,301)	69%	80%	75%	76%	1.52E-08

Diff, differential expression between the two miRNAs, calculated as the difference between Ct values of the two indicated miRNAs. PPV, positive predictive value; NPV, negative predictive value; SENS, sensitivity; and SPEC, specificity. The cutoff value used in training set was applied in the test set.

## Discussion

In this exploratory Phase I/II biomarker study (as outlined by the Early Detection Research Network (EDRN) (http://edrn.nci.nih.gov)) we screened serum from patients with no history of cancer, and patients with NSCLC in an effort to identify miRNAs that can be used as biomarkers for the detection of early stage lung cancer. We identified a miRNA pair miR-15b/miR-27b that was able to distinguish between serum from NSCLC patients and cancer-free healthy controls, and with a high degree of sensitivity. Our results support the findings of recent studies that have shown that circulating miRNAs profiles may be useful in screening for NSCLC, [16], [17] however, our study included substantially more patients (180 total) than either of these previous studies. One drawback of these biomarkers is the low specificity of 84% in the test set. The relatively high false positive rate for these tests could be considered unacceptable for screening the general population. However, in a population of smokers at high risk for lung cancer, unnecessary screening would be mitigated by the ability of this test, with its negative predictive value of 100%, to be able to exclude a large number of patients from going on to more expensive screening modalities, such as helical chest CT. Although these data are compelling, further testing in a large, prospective cohort as a Phase III biomarker study is required to assess the clinical utility of these miRNA markers as a first line screening test for NSCLC.

The miRNAs markers identified in this study have previously been implicated in human malignancies. miR-15a and miR-15b have been shown to be de-regulated in human lung cancer [21]. Both miR-15a and miR-15b have been shown to have a diagnostic and prognostic value in chronic lymphocytic leukemia [22], [23]. miR-27b was found to be down-regulated in lung cancer tissues compared to non-cancerous lung tissue [24]. In addition, miR-27b expression levels have been correlated with invasiveness of breast cancer [25] and with regulation of angiogenesis [26]. A study involving a total of 86 NSCLC and 57 controls recently revealed a four miRNA panel in plasma including miR-126 that distinguished NSCLC from healthy controls with a sensitivity and specificity of 73% and 96% respectively [27]. Using whole blood, Keller and coworkers [28] showed that miR-126 and miR-98 were among the top miRNAs that could distinguish NSCLC from healthy controls. miR-126 is highly expressed in lung tissue and is involved in the regulation of vascular cell adhesion molecule 1 (VCAM-I) [29]. Aberrant expression of miR-126 has been implicated in the pathogenesis of NSCLC [30], [31]. In this study, several pairs involving miR-126 were among the several candidates identified in the training set (Figure S1), however upon further testing in additional 130 samples, all the miRNA-126 candidate pairs exhibited sensitivity greater than 75% and the specificity was less than 75%. Other recent studies by Foss and coworkers [17] showed serum miR-1254 and miR-574-5p were differentially expressed between NSCLC (n = 33) and healthy controls (n = 42). It is important to note that several factors affect the outcome of miRNA studies in biofluids, including variations in sample type e.g. whole blood, plasma or serum, sample numbers, study design, sample collection, RNA isolation, patient characteristics, number of miRNA examined and technologies used in miRNA profiling e.g. solexa sequencing, RT-qPCR or microarray technologies. In addition, standardization of isolation methods, normalization and data analysis methods is needed in order to demonstrate a clear clinical utility of these putative markers.

Although these data are promising, the test set included a relatively small number of samples. Ultimately, these miRNA biomarkers require further validation on larger prospective cohorts such as a Phase III biomarker study in order to validate these results. Incorporating blood-based miRNA markers in spiral CT studies may aid in exploring the utility of miRNAs in screening of lung cancer. Although this is an exploratory phase I/II trial, the patients were selected primarily from surgical clinics, and are weighted towards early stage disease (60% Stage I, 24% Stage II, 12% Stage III and 4% Stage IV). This skew towards early stage disease supports the investigation of these markers in a phase III or IV trial aimed at defining the performance of these markers in a prospective manner in early stage detection.

The recent dissemination of the utility of screening helical chest CT scans for reduction in mortality from lung cancer from the NLST trial places a premium on identification of high risk individuals who could benefit from screening. Adjunctive serum based testing may be performed in a highly cost effective manner compared to imaging, and may be helpful to identify high risk populations that may benefit from chest CT, or to be used in combination with imaging to identify early lung cancers.

There is great need for improved screening for lung cancer given the large number of people affected each year and the high mortality rate of the disease when diagnosed in its later stages. There are currently numerous clinical trials being conducted to test the efficacy of novel therapies for NSCLC, however, the majority of these are Phase II trials and recently a number of Phase III trials have failed to meet their primary end points [32] To date, improved screening to provide early detection is the most promising avenue to reduce mortality from NSCLC. Our study further strengthens the argument that serum miRNA have the potential to be used as a cost effective, non-invasive diagnostic test for NSCLC, and could potentially be used as a first line screen to help risk stratify patients for further, more expensive or invasive screening regimens.

## Supporting Information

Figure S1 **Differential miRNA expression in sera from NSCLC patients (cancer) and healthy controls (normal) in training set.** Diff, differential expression between the two miRNAs, calculated as the difference between Ct values of the two indicated miRNAs. PPV, positive predictive value; NPV, negative predictive value; SENS, sensitivity; and SPEC, specificity. The cutoff value used to achieve the indicated specificity and sensitivity is indicated for each miRNA diff pair.
(XLS)

## References

[1] Jemal A, Bray F, Center MM, Ferlay J, Ward E, et al. (2011). Global cancer statistics.. CA Cancer J Clin.

[2] Centers for Disease Control and Prevention (CDC) (2007). Cigarette smoking among adults–United States, 2006.. MMWR Morb Mortal Wkly Rep.

[3] Henschke CI, Yankelevitz DF, Libby DM, Pasmantier MW, Smith JP (2006). Survival of patients with stage I lung cancer detected on CT screening.. N Engl J Med.

[4] Aberle DR, Berg CD, Black WC, Church TR, Fagerstrom RM (2011). The National Lung Screening Trial: overview and study design.. Radiology.

[5] New York Early Lunc Cancer Action Project Investigators (2007). CT Screening for lung cancer: diagnoses resulting from the New York Early Lung Cancer Action Project.. Radiology.

[6] Peltier HJ, Latham GJ (2008). Normalization of microRNA expression levels in quantitative RT-PCR assays: identification of suitable reference RNA targets in normal and cancerous human solid tissues.. RNA.

[7] Bach PB, Jett JR, Pastorino U, Tockman MS, Swensen SJ (2007). Computed tomography screening and lung cancer outcomes.. JAMA.

[8] Miettinen OS (2000). Screening for lung cancer: can it be cost-effective?. CMAJ.

[9] Welch HG, Black WC (2010). Overdiagnosis in cancer.. J Natl Cancer Inst.

[10] Welch HG, Woloshin S, Schwartz LM, Gordis L, Gotzsche PC (2007). Overstating the evidence for lung cancer screening: the International Early Lung Cancer Action Program (I-ELCAP) study.. Arch Intern Med.

[11] Russi EW (2011). Lung cancer screening has the potential to safe lives, but shall we do it?. Swiss Med Wkly.

[12] Brenner DJ (2004). Radiation risks potentially associated with low-dose CT screening of adult smokers for lung cancer.. Radiology.

[13] Bellomi M, Rampinelli C, Funicelli L, Veronesi G (2006). Screening for lung cancer.. Cancer Imaging.

[14] Aggarwal C, Somaiah N, Simon GR (2010). Biomarkers with predictive and prognostic function in non-small cell lung cancer: ready for prime time?. J Natl Compr Canc Netw.

[15] Mitchell PS, Parkin RK, Kroh EM, Fritz BR, Wyman SK (2008). Circulating microRNAs as stable blood-based markers for cancer detection.. Proc Natl Acad Sci U S A.

[16] Boeri M, Verri C, Conte D, Roz L, Modena P (2011). MicroRNA signatures in tissues and plasma predict development and prognosis of computed tomography detected lung cancer.. Proc Natl Acad Sci U S A.

[17] Foss KM, Sima C, Ugolini D, Neri M, Allen KE (2011). miR-1254 and miR-574-5p: serum-based microRNA biomarkers for early-stage non-small cell lung cancer.. J Thorac Oncol.

[18] Lin PY, Yu SL, Yang PC (2010). MicroRNA in lung cancer.. Br J Cancer.

[19] Komatsu S, Ichikawa D, Takeshita H, Tsujiura M, Morimura R (2011). Circulating microRNAs in plasma of patients with oesophageal squamous cell carcinoma.. Br J Cancer.

[20] Szafranska AE, Davison TS, John J, Cannon T, Sipos B (2007). MicroRNA expression alterations are linked to tumorigenesis and non-neoplastic processes in pancreatic ductal adenocarcinoma.. Oncogene.

[21] Bandi N, Zbinden S, Gugger M, Arnold M, Kocher V (2009). miR-15a and miR-16 are implicated in cell cycle regulation in a Rb-dependent manner and are frequently deleted or down-regulated in non-small cell lung cancer.. Cancer Res.

[22] Calin GA, Ferracin M, Cimmino A, Di Leva G, Shimizu M (2005). A MicroRNA signature associated with prognosis and progression in chronic lymphocytic leukemia.. N Engl J Med.

[23] Calin GA, Liu CG, Sevignani C, Ferracin M, Felli N (2004). MicroRNA profiling reveals distinct signatures in B cell chronic lymphocytic leukemias.. Proc Natl Acad Sci U S A.

[24] Yanaihara N, Caplen N, Bowman E, Seike M, Kumamoto K (2006). Unique microRNA molecular profiles in lung cancer diagnosis and prognosis.. Cancer Cell.

[25] Wang Y, Rathinam R, Walch A, Alahari SK (2009). ST14 (suppression of tumorigenicity 14) gene is a target for miR-27b, and the inhibitory effect of ST14 on cell growth is independent of miR-27b regulation.. J Biol Chem.

[26] Kuehbacher A, Urbich C, Dimmeler S (2008). Targeting microRNA expression to regulate angiogenesis.. Trends Pharmacol Sci.

[27] Shen J, Todd NW, Zhang H, Yu L, Lingxiao X (2011). Plasma microRNAs as potential biomarkers for non-small-cell lung cancer.. Lab Invest.

[28] Keller A, Leidinger P, Borries A, Wendschlag A, Wucherpfennig F (2009). miRNAs in lung cancer - studying complex fingerprints in patient's blood cells by microarray experiments.. BMC Cancer.

[29] Harris TA, Yamakuchi M, Ferlito M, Mendell JT, Lowenstein CJ (2008). MicroRNA-126 regulates endothelial expression of vascular cell adhesion molecule 1.. Proc Natl Acad Sci U S A.

[30] Sun Y, Bai Y, Zhang F, Wang Y, Guo Y (2010). miR-126 inhibits non-small cell lung cancer cells proliferation by targeting EGFL7.. Biochem Biophys Res Commun.

[31] Liu B, Peng XC, Zheng XL, Wang J, Qin YW (2009). MiR-126 restoration down-regulate VEGF and inhibit the growth of lung cancer cell lines in vitro and in vivo.. Lung Cancer.

[32] Subramanian J, Madadi AR, Dandona M, Williams K, Morgensztern D (2010). Review of ongoing clinical trials in non-small cell lung cancer: a status report for 2009 from the ClinicalTrials.gov website.. J Thorac Oncol.

