# Correction: Serum microRNA Biomarkers for Detection of Non-Small Cell Lung Cancer

**DOI:** 10.1371/annotation/bda2b1d8-1054-481a-bafd-2bcfa48514e6

**Published:** 2012-05-29

**Authors:** Patrick T. Hennessey, Tiffany Sanford, Ashish Choudhary, Wojciech W. Mydlarz, David Brown, Alex Tamas Adai, Michael F. Ochs, Steven A. Ahrendt, Elizabeth Mambo, Joseph A. Califano

There were errors in the Author Contributions. The correct contributions are: Conceived and designed the experiments: JC, EM, PH, DB.

Performed the experiments: TS.

Analyzed the data: AC, ATA, MO.

Contributed reagents/materials/analysis tools: PH, WM, SA.

Wrote the paper: PH, EM, JC, AC. 

